# Spectrum of complaints: practices of complaining in therapeutic conversations as a window to spouses' personalities and couples' relationships

**DOI:** 10.3389/fpsyg.2023.1232594

**Published:** 2023-11-23

**Authors:** Bartłomiej Taurogiński, Bernadetta Janusz, Jörg R. Bergmann, Anssi Peräkylä

**Affiliations:** ^1^Laboratory of Systemic Psychology and Psychotherapy, Jagiellonian University Medical College, Kraków, Poland; ^2^Department of Family Therapy and Psychosomatics, Jagiellonian University, Medical College, Kraków, Poland; ^3^Faculty of Sociology, Bielefeld University, Bielefeld, Germany; ^4^Faculty of Social Sciences, University of Helsinki, Helsinki, Finland; ^5^Freiburg Institute for Advanced Studies, University of Freiburg, Freiburg, Germany

**Keywords:** conversation analysis, psychotherapy, couple therapy, complaining, blaming

## Abstract

**Introduction:**

Complaining is a frequent phenomenon in human interactions and it frequently happens during couple counseling. A conversation between a therapist and spouses that requires them to talk about problems inevitably leads to complaining (especially during the first meeting). The institutional context and the presence of an impartial therapist shape the complaining sequences.

**Method:**

We used conversation analysis to explore the interactional organization of complaining in the specific context, which is couples therapy. Our data involve video recordings of nine couple therapy first consultations.

**Results:**

In the results section of our paper, we describe in detail the composition and delivery of complaints in couple therapy setting. Our observations made it possible to propose a nuanced spectrum of ways of complaining that spans the considerateness dimension. Our data suggest that there may be a relationship between the manner of complaining and the presence and severity of maladaptive personality traits of complainers.

**Discussion:**

We argue that paying close attention to complaining practices that arise during couple therapy is an important aspect of clinical work with couples and can be informative regarding the nature of spouses' quarrels and their personality constitutions.

## 1 Introduction

Problem formulation is a constitutive feature of any professional–client encounter (cf. Pino and Mortari, 2012). Clients need to describe early on the reason why they look for professional support or help. One of the specific contexts for getting help and talking about problems is couples therapy, which involves a complex and delicate triadic constellation (cf. Stivers and Majid, 2007; Stivers, 2012), where clients formulate their marital problems and their complaints for the therapist as the addressed recipient in the presence of the unaddressed but overhearing spouse who is the target of the complaint (cf. Wilkinson et al., 2013). This seems to be vital, particularly in the context of first consultations. The way spouses complain in such a peculiar context is the focus of the following study.

When couples embark on couple therapy, they usually do so to find a solution for their marital problems. For this, the spouses routinely unfold these problems during the first consultation. However, compared with other types of professional–client interaction, formulating “the problem” in a couple therapy setting is characterized by a unique constellation. Here, both spouses are virtually part of “the problem.” Any description of the problem by one spouse can include the respective partner or make implicit or explicit reference to him/her. Even neutral descriptions of subjective annoyances or the couple's insufficient functioning may involve the partner and can be hearable as a covert complaint. Moreover, even the question of the nature of the marital problem or whether there is a problem at all may be contentious among the spouses. Spouses who come to therapy usually have a long history of mutual complaining, blaming, and marital disputes. It is a general observation that these quarrels tend to be re-enacted in the therapeutic setting and that clients tend to locate responsibilities, marital problems, and their causes in the other spouse.

### 1.1 Complaining in couple therapy—Clinical practice and clinical research

Complaining, accusing, and blaming are actions that frequently occur in therapeutic conversations, especially at the beginning of the therapy process. They are closely linked to taking a defensive or offensive position, hindering reflection and ultimately making it difficult to solve problems. They are high-impact attributions that can provoke shame, guilt, anger, or some other aversive emotional state (Friedlander et al., 2000).

In empirical psychotherapy research, some attention has been paid to the verbal forms and social dynamics of complaining as a conversational activity within a triadic interactional constellation. Research was done on the activity of complaining/blaming itself, reactions of others to complaining/blaming, and how therapists deal with this phenomenon in their practice. For example, Stratton (2003a,b) analyzed attributions of responsibility and blame in family therapy using a special, manual coding system. He pointed at “characterological blaming” as the most damaging form of blame and suggested sensitizing therapists to this phenomenon. Some authors studied blame and accusation as elements of a moral discourse in which responsibility is topicalized and negotiated in marital and couple therapy (Buttny, 1990; Edwards, 1995; Kurri and Wahlström, 2005). Others (Beck, 1987; Friedlander et al., 2000) pursued the response of spouses and therapists following blame expressed by family members and singled out different dimensions in the therapists' actions, including their “neutral” stance (Stancombe and White, 2005). O'Reilly (2005) examined episodes in family therapy during which a client complains to the therapist about a non-present third party (an agency or an individual). She showed that complaints are made by constructing something as negative, attributing moral fault, and assigning agency/responsibility. Although she furthermore observed that the therapist's responses display an orientation toward the “unhelpfulness of complaints,” she disregarded how the other co-present clients participate or are involved.

Ways of complaining are intertwined with the personalities of the clients. Since complaining and being complained about is an emotionally challenging interaction (Päivinen et al., 2016), one may say that it is particularly difficult for individuals struggling with regulating their emotions, which is postulated to be a core clinical symptom of personality disorders (PDs) (Livesley and Larsone, 2018). Literature on therapy with personality-disordered couples is still scarce and is mainly concerned with how such persons present themselves in therapy and how to adapt the way of conducting therapy to specific personality styles (McCormack, 2000; Landucci and Foley, 2014). Although there are descriptions of different ways in which spouses with specific psychological problems complain in couple relationships (Lachkar, 2014), in no articles to date have authors paid attention to the details of complaining practices and their interactional relevance in the context of therapy with personality-disordered couples.

### 1.2 Complaining from a conversation analytic perspective

To get a deeper understanding of complaining practices in couple therapy, a more sophisticated observational perspective is called for. As we have done in earlier research (Janusz et al., 2021; Peräkylä et al., 2023), we adopt a conversation analytic approach which enables us to identify conversational details of complaining activities as well as sequences in which clients prepare and deliver a complaint or respond to it. A conversation analytic perspective on complaining practices in marital therapy is justified because complaints, accusations, and similar ways in which interlocutors deal in everyday life with a deemed wrongdoing are a prominent topic within that research tradition.

In everyday life, “complaining” and its meaning is an unquestioned matter of course. It is, however, difficult to find an exact formal definition for this activity (Pillet-Shore, 2015). Complaining, blaming, and accusing are common-sense concepts, and as such they are essentially vague; their meanings blend into each other, but all of them refer to the display of some negative experience or stance. Although without clear cut demarcations, complaints and related phenomena can be differentiated and ordered in a sequence along their social and moral design. The most neutral way of expressing some pain or the feeling of discontent about some personal mishap is what we call Jefferson's (2015) “*trouble telling*.” Trouble can be communicated by moaning, such as when some annoyance is expressed without reference to any cause or culprit. *Complaining*, on the other hand, refers more specifically to the display of some suffering or negative experience, but in complaining, responsibility is either sought or can be attributed to “someone” (Heinemann and Traverso, 2009). *Blaming* is a morally charged form of complaining and is outward-oriented to an identified perpetrator. Compared to complaining and blaming, *accusing* is the most inconsiderate and offensive way of displaying indignation. Accusing is realized as a moral attack and captures a strong way of charging a person directly and quite often by non-verbal means of some infringement. The wrongdoer is known and is confronted with the speaker's negative experience and his/her supposed social or moral violation (Castor, 2015).

When the interaction is triadic, complaining becomes interactionally more complex. In such a participation framework, the distinction between the recipient and the target of a complaint is pertinent. A speaker can aim the complaint directly at the co-present addressee, where the recipient and the complaint's target are identical. However, the recipient of a complaint need not be the complaint's target. In contrast to *direct complaints*, in which the recipient is directly made responsible for the speaker's discontent, *indirect complaints* are characterized by the fact that the deemed culprit is either not present in the interaction or, if there, is not addressed. In triadic or multi-party conversations, co-present unaddressed third parties can take sides and can—by commiserating—even affiliate with the complainer (Boxer, 1993), which in effect “collectivizes” the complaint (Laforest, 2009) and often prevents the complaint target from defending him/herself (Heinemann, 2009). For example, in couple therapy, a client can witness how his/her spouse attempts to win the therapist over on his/her side, thus partitioning the conversation and building a complaint coalition against the bypassed partner.

In addition to the relational framework and the associated distinction between direct and indirect complaints, three other dimensions must be mentioned along which modes of complaining can be distinguished from each other. Complaints differ with regard to their *affective* intensity, which may range from a cool slight to a heated allegation. Second, complaints have a *developmental dimension* and can evolve—often in response to the recipient's first reactions—from a seemingly innocent and neutral observation to an offensive charge or from an inconsiderate blaming to a subdued criticism. Third, complaints are also potentially *face-threatening* (Goffman, 1955), and their realization can differ with regard to the degree to which a speaker takes aspects of face-saving into account.

## 2 Objective

The study we present is part of our ongoing research on the therapeutic conversation in couples therapy with participants with personality disorder traits. Focusing on conversational segments in which the couple was asked to formulate the marital problem to be solved in therapy, episodes in which one spouse complains directly or indirectly about the other are singled out and analyzed in close empirical detail. Against the background of the structural features of complaining outlined above, our main objective is to distinguish and identify practices and typical “styles” of complaining about the spouse in couple therapy and to arrange them in a spectrum that encapsulates the differences between them. We are convinced that the specific ways in which spouses complain about each other may not only illuminate their specific marital relationship but may also be related to their individual personality traits and capabilities of managing close relationships and regulating emotions.

The rationale of our study is that the way in which a complaint is made makes reference to the spouse and is answered by the spouse, which is an important indicator for the therapist. The success of therapeutic efforts to move the spouses' talk away from blame game and to reach a productive mode to work with their problem not only depends on the therapists' attitudes and their professional skills but also on their knowledge of the phenomenology of complaining in couple therapy. We furthermore pursue the idea that couple and family therapists, irrespective of their therapeutic school, can enhance their professional competence by increasing their sensitivity to the client's expressions of blame or accusation and the client's emotional responses to moral actions of this kind.

## 3 Materials and methods

### 3.1 Data collection

The data were collected as part of the wider research project called “Facing Narcissism” (https://www.helsinki.fi/en/researchgroups/narcissism-face-and-social-interaction/studies). One branch of this project is a study of therapeutic conversations of couples where one spouse has traits of narcissistic (or other) personality disorder (therefore, the decision to include material in the database was driven by clinical features of the interactants). Researchers decided to use material from couples therapy conducted at the Family Therapy and Psychosomatics Department, Jagiellonian University Medical College in Cracow, Poland, where couple therapy sessions are routinely video recorded for supervision and training. The project got the agreement of the Bioethical Committee, Jagiellonian University Medical College no. 1072.6120.76.2020. The participants gave written consent to using video recordings of their interactions for current research.

The couples were recruited to the project in two phases. First, therapists from the department were asked to make, among the couples that they had worked with, an initial clinical judgment and identify those couples where at least one of the spouses presented symptoms of personality disorder. The decision to include a couple in the study was made after they had finished couple therapy; thus, their reason to start therapy was in no way related to the decision to participate in the current study. In the second phase, a member of the research team (BJ) selected couples to be assessed in more detail with the Shedler-Westen Assessment Procedure (SWAP; Shedler and Westen, 2007) and after that selected the cases to be included in the database. The exclusion criterion was the presence of other psychopathology than PD (such as psychotic symptoms or bipolar disorder). For comparative purposes, we decided to include two couples without personality pathology. They were also assessed with SWAP to confirm the lack of such features.

SWAP is an instrument used for making personality assessments and is completed by the clinician who has worked with the patient for some time, not the patient. The result of this procedure is a personality profile of the subject. It is important to note that therapy sessions that make up our database were conducted before the therapists filled out the SWAP questionnaire.

Consultations were conducted following integrated systemic and psychodynamic approaches. According to them, therapists, during first consultations, aim to find out how each spouse defines “the problem” and what husband and wife expect from therapy (Stierlin et al., 1980). Defining the couple's problem may already lead to complaints or blaming during the consultation, which is why the spouses are expected to talk directly to the therapist (not to each other) to prevent the upcoming of their dysfunctional relational patterns (Sprenkle and Blow, 2007).

### 3.2 Participants

Our database consists of video recordings of initial consultations of nine couples that were conducted by eight different psychotherapists. Spouses were manifesting diverse personality styles—in seven couples, at least one of the spouses was assessed as having personality disorder features, while in two couples, no personality disorder traits were found. The researchers involved in the project worked with the entire available data. However, for the ensuing presentation of our study, we decided to present extracts from sessions of four different couples conducted by four different psychotherapists. Two of the presented couples were not characterized by personality pathology, and in the other two, spouses were assessed as having traits of either borderline or narcissistic personality disorder. The possible connections between personality pathology and complaining practices will be taken up in the discussion (Section 6.2).

### 3.3 Conversation analysis

The data analysis in our project did not start from the couples' clinical assessment but followed the bottom-up approach of conversation analysis in the identification of different practices of complaining. It involved (1) extracting all segments from the nine sessions that contained complaining or blaming extracts (according to the definitions described in Section 1.2); (2) unmotivated data analysis of all complaining extracts; (3) identifying more considerate and inconsiderate complaining practices; and (4) choosing four couples that represent the spectrum of complaints from the most considerate to the most inconsiderate.

Conversation analysis (CA) is a social research methodology for the detailed analysis of naturally occurring talk-in-interaction that is audio- or video-recorded and then transcribed with a standardized system of orthographical transliteration and additional suprasegmental markers (Peräkylä et al., 2008; Sidnell and Stivers, 2013). CA studies pay particular attention to the sequential organization of social interaction; however, it also developed methods for the analysis of descriptive practices with which single turns are constructed, objects are formulated, or events are described. Early studies have shown how extreme case formulations (Pomerantz, 1986), idiomatic expressions (Drew and Holt, 1988), “negative observations” (Schegloff, 1988), or the rhetoric device “litotes” (Bergmann, 1992) are applied to mark the moral implications of an utterance.

CA methods have been used to study many types of conversations in non-institutional and institutional contexts, including family conversations, social chats, medical interviews, and mediated communication. For some years, conversation analysis has also been applied to the study of the psychotherapy process, allowing researchers to micro-analyze recorded psychotherapy sessions (Peräkylä, 2008). Studies of psychotherapeutic interaction have so far focused on issues such as formulations (Antaki, 2008), interpretations and responses to them (Peräkylä, 2011), questions (MacMartin, 2008), resistance (Vehviläinen, 2008), affiliation (Muntigl and Horvath, 2014), and expression of emotion (Leudar et al., 2008). Most of this research was conducted in the context of individual psychotherapy, that is, in the context of a dyadic interaction. Some CA studies have dealt with multi-party interaction (Lerner, 1996; Fioramonte and Vásquez, 2019), but studies of triadic psychotherapeutic settings are rare. In their comprehensive review of studies utilizing CA as a methodology for the analysis of family therapy, Ong, Barnes, and Buus (Ong et al., 2020) found only 25 studies, which document the emerging interest in CA research in this area.

### 3.4 Data analysis

The data analysis initially involved unmotivated exploration aimed at recognizing interactional practices in couples with or without personality disorders. This led to more focused work on phenomena such as “controlling the interaction” (Janusz et al., 2021) and “disengagement in the interaction” (Peräkylä et al., 2023). The current project was focused on complaining practices. We extracted segments from the nine sessions that contained complaints (according to the definitions described in Section 1.2), and then we started descriptive data analysis of all these extracts. This made it possible to identify more considerate and inconsiderate complaining practices. Finally, we chose five extracts from four couples representing the spectrum of complaints from the most considerate to the most inconsiderate.

## 4 Results

Our overall impression about the data is that ways of complaining differ across couples: each couple may have their characteristic ways of complaining. In the following, we provide examples of complaining talk of four different couples. The sequential order in which the complaining practices are presented is based on our observation that these practices can be arranged according to the degree to which the spouses consider their respective partners in their complaints. The analysis starts with an extract from a couple in which the spouses complain about each other in the most cautious ways. We then present extracts from complaining in other couples. In these extracts, the degree of inconsiderateness increases so that the last extract shows a couple where the spouses mutually blame each other directly. Thus, the succession of the extracts in the following analysis documents that the manifold practices of couples to mutually criticize or morally attack each other in couples therapy form a spectrum of complaints.

Presented extracts are in Polish. Considering the Polish cultural context, the most expected form of referring to a spouse is to use the phrase “husband/wife/partner” or “my husband/wife/partner.” It should be noted that in Polish, there are no prepositions, such as the English “the”, “a”, and “an.” Sometimes, the use of the pronoun “he/she” alone in the presence of the subject of the sentence can indicate the building of relational distance. It is also not typical for spouses to use their partners' names when describing their behavior at the first meeting with the therapist. The use of a spouse's name in a statement can mean shortening the distance with the therapist or not including him or her at all in the context of the conversation at hand (as one can see in Couple 4).

### 4.1 Couple 1: strict mother

*During the talk with the therapist about family problems, the wife described difficulties in disciplining the younger child and conveyed in a complaint-implicative way that in her view, her husband is too lenient with the children, leaving the task of disciplining to her. In a mitigated way, she conveys that she once hit one of the children. She described the situation as a reversal of the parental roles of father and mother and mentioned her difficulties with this role reversal and her own problematic behaviors. After the wife described the problem from her perspective, the therapist turned to the husband (H) and asked him to present his point of view. In his elaborate answer, H gives his view of the situation, which implies a (counter-) complaint about his wife:*

**Extract 1a**

07 H: hhh znaczy, tak, wydaje mi sie (.) moze to nie jest, ze ze zona jest
**hhh I mean, well, it seems to me (.) maybe it isn't, that that wife**
08 stanowcza tylko ze: (0.5) e e no: (1.0) bo to ja bo ja bo (4.0)
**is strict but tha:t (0.5) um um well: (1.0) because I because I (4.0)**
09 ucieka w sytuacjach w których stanowczość sie przeradza juz
**he runs away in situations when strictness transforms**
10 (1.5) juz czasami (1.0) >tak jak zona powiedziała< zda- zdarza sie to
**(1.5) sometimes even (1.0) >as wife said < it ha- happens**
11 bardzo rzadko .hh ale przeradza sie juz w jakieś:: (1.0) odrzucenie
**very rarely .hh but it transforms into kind of:: (1.0) rejection**

H starts his account of the marital problem by correcting his wife's (W) prior description of her difficulties with disciplining the younger child, pointing out that the issue is not W's “strictness” but the fact that strictness sometimes turns into rejection. “Rejection” of a child is understood as inappropriate parental behavior, which makes H's account hearable as a complaint about his wife's behavior. However, H produces the account in a cautious and considerate way. He furnishes the account with uncertainty markers (line 7: “maybe it isn't” and line 11: “kind of”), produces self-repair (line 08: “because I- because I”), and mitigates the account by pointing out that his wife's problematic behaviors are infrequent (line 11). Furthermore, he conveys deference to his wife's prior report of the situation by presenting his agreement with it (lines 10–11: “as the wife said it happens very rarely”).

After having characterized his wife's problematic behavior, H moves on to depict the situation from his own perspective, describing his own way of handling it. He starts by claiming his own helplessness, pain, and the child's confusion:

**Extract 1b**

14 H:.hhh >ja w takiej sytuacji< nie wiem jak reagować.
**.hhh >in such situation< I don't know how to respond**.
15 szczerze mówiac. bo, bo, bo mi jest przykro, a tez
**to be honest. because, because, because I'm hurt, and also**
16 (0.5) DZIECKO płacze, nie wie co sie stało.
**(0.5) THE CHILD is crying, it doesn't know what has happened**.
17 T: **Mhm**.

This description of helplessness extends and intensifies H's complaint. The description clearly implies that W's way of 'rejecting' the child is causing confusion and suffering; yet, H does not (at this point) mention his wife's behavior, focusing instead on his and the child's suffering.

After the continuer (line 17) from the therapist (T), H elaborates the description of his helplessness in finding the right way to respond to the child's crying. He presents two alternative ways of acting, which for him both feel wrong: holding the child (thereby showing himself as the “loving” parent) or pushing it away (transcript not included). The elaboration focuses solely on the child and the husband himself; yet, the account implies that the child is crying because of the conflicts with the wife.

In response to H's description of his helplessness, T provides a formulation (lines 23 and 25) that preserves the topical focus on H's own behavior (rather than on W's actions):

**Extract 1c**
22 T: mhm (.) to mówi pan o takiej trudności pogodzenia
**well you are talking about the difficulty**
23 .hh [ ] (.) dania wsparcia zonie
**.hh [ ] (.) of giving wife support**
24 H:[tak]
**[yes]**
25 T:i zainteresowania [sie dzieckiem?
**and showing interest [to your child?**
26 H:[do- do- dokładnie staniecia po stronie zony
**[ex- ex- exactly to take wife's side**
27 bo bo bo zawsze staram sie >stanać po stronie zony<.hh
**cause cause cause I always try to >take my wife's side< .hh**

As soon as T formulated H's interest in supporting his wife, H confirms T's formulation in overlap (lines 26 and 27). H's early and emphasized confirmation also forestalls a reading of his prior description as one that is primarily a complaint about his wife, and the reading of his sentiment or motivation being that of complaining.

Yet, after emphasizing his willingness to take his wife's side, H continues his response and returns to talk about her complainable behavior (see Extract 1e below). By disclosing that he does not accept hitting (line 28), he indirectly brings to the topical focus the fact that W was hitting the child. However, he presents the rejection of hitting as a joint decision or policy of the couple (lines 28, 29, 32–34). Thereby, he includes, as it were, his wife in a “team” that is against W's complainable behavior. Thus, H protects his wife's self in a situation where he is discussing episodes in which she indeed has hit the child. Interestingly, in the utterance where H for the second time includes his wife in the anti-hitting collective, he does self-repair from the first-person singular to first person plural (lines 32–33: “and I adopted a principle (.) that: (1.0) we decided together”); the self-repair indicates H's hesitation in talking about the matter.

**Extract 1d**
28 H:nie toleruje bicia i to ześmy przy- przy- przyjeli >taka zasade
**I don't tolerate hitting and we ad- ad- adopted >such a common**
29 wspólna< od ZAWSZE .hhh hhhh (1.5) byłem bardzo rzadko bity, przez
**principle< since FOREVER .hhh hhhh (1.5) I was beaten very rarely, by**
30 rodziców. a pamietam to (0.5) .hhhhh kazda sytuacje.
**parents. and I remember it (0.5).hhhhh each incident**.
31 T: mhm
32 H: e:: i przyjałem zasade (.) ze: (1.0) ze ustalaliśmy
**eh:: and I adopted a principle (.) that: (1.0) we decided**
33 wspólnie ze ze ze >ze nie bijemy< dzieci i ze ze
**together that that that >we do not hit < children and that that**
34 nie podniesiemy reke na dziecko .hhh i reaguje (.) złościa
**we won't raise a hand on child .hhh and I respond (.) with anger**
35 w sytuacji w której widze ze: (1.5) jes jestem zdenerwowany
**in situation in which I see that: (1.5) I- I am angry**
36 i i broń Boze do (.) nie chodzi o jakieś rekoczyny ale
**and and God forbid (.) it's not that there are some physical but**
37 pokazuje swoje emocje, (.) ze bardzo mi sie nie podobaja takie
**I show my emotions, (.) that I very much dislike such**
38 zachowania. Pokazuje to n:: dzieci myśle ze to tez widza.
**behaviors. I show this n:: I think that children see this as well**.

After having described the couple's principle not to hit children, H moves on to depict more concretely his reaction to hitting (lines 34–38). The account focuses primarily on his own feelings and actions and on the ways in which children see his reaction. What he reacts to is described in very indexical terms, as “that” (line 35) and “such behaviors” (lines 37–8). By choosing these oblique terms, H seems to avoid references to his wife's problematic behavior (i.e., hitting). On the other hand, by describing his anger when he sees “such behaviors,” H presents himself as a moral person who reacts to wrongdoings and wants to protect the children.

After a short repair sequence, H continues his account, now focusing on the children's perception of the problematic family scenes (lines 43–48). As H is seeking to capture what the younger child sees (line 48), his wife cuts in with a rewording confirmation “he registers. yes” (line 49). By offering her confirmatory rewording, W accepts and participates in her husband's depiction of the problematic family scene. Thereby, she treats herself and her husband as belonging to the same social and experiential unit. In so doing, W at this moment ratifies her husband's account and implicitly admits that her behavior may be problematic.

**Extract 1e**
43 H: tak. (1.0) myśle ze dzieci to tez widza (.) nie wiem
**yes. (1.0) I think that children also see it (.) I don't know**
44 jak córa, bo córa z reguły
**about daughter, because daughter usually**
45 w w:: tym nie uczestniczy
**doesn't participate in i::n this**
46 bo ona sie gdzieś tam bawi,
**because she plays somewhere**,
47 ale ale .hh myśle ze mały to gdzieś
**but but .hh I think that the little-one sees it**
48 katem oka (0.6) er::m widzi, ze=
**with the corner of his eye (0.6) er::m sees that=**
49 W: rejestruje. [tak.
**he registers. [yes**.
50 H: [rejestruje ze ze z- tata jest niezadowolony
**[registers that that th- daddy is not happy**
51 ze ze ze mama (.) mama mama uderzyła, (0.4)
**that that that mother (.) mother mother hit, (0.4)**
52 i gdzieś moze gdzieś tam w głebi psychiki (.) widzi
**and somewhere maybe somewhere deep in his psyche (.) sees**
53 ze ze ze tata stoi (.) po jego stronie.
**that that that daddy is (.) keeping his side**.

H confirms his wife's rewording “register” (line 50) and continues with an explication of what the child saw (lines 51–52). In describing the child's perception—that “*daddy is not happy that mother hit*”—H eventually conveys a most severe complaint that is deeply threatening for the self of his wife. The description “*mother hit*” is produced in a particularly considerate way. It is not only delayed by serial repetitions of “*that*” and “*mother*” (line 51), but syntactically, the clause “*mother hit*” is nested in several other clauses: “*the little one* (...) *registers*” (lines 47, 50), “*that daddy is not happy*” (line 50), and “*that mother hit*” (line 51). Noticeably, the most “damaging” description is followed by silence (line 54). Continuing his utterance (line 52), H reflects on the child's perceptions, thus moving topically away from his wife's actual behaviors. By this topical shift in the continuation of his utterance, and through the nesting of his assertion of his wife's behavior, H softens and downplays the message “*mother hit*.”

After H has completed his account, T takes the turn and asks W to comment on what her husband has just said (line 54).

**Extract 1f**

54 T:mhm. a pani jak rozumie taka sytuacje
**mhm. and you(f) how do you understand such situation**
55 kiedy dochodzi .hh do takich e:rm
**when it comes .hh to such e:rm**
56 tez erm róznic rozumiem miedzy państwem w podejściu?
**also erm differences as I understand in your approach?**
57 H: chciałem powiedzieć tylko jeszcze jeszcze sie wtrace
**I wanted to say I wanted to add only**
58 ze to [była< .hh przez ostatni rok to była (.) to było kilka razy
**that it [was < .hh over the last year (.) such incidents**
59 T: **[aha**
60 H:to sie to sie nie dzieje ze to jest
**were only few times it doesn't it doesn't happen that**
61 ze zona leje dzieci[: dziennie tam pasem
**that wife hits children[: daily with a belt**
62 W:**[£TcHhh£**
63 H: to to to było moze nie wiem pieć razy, sześć razy
**it it it happened maybe I don't know five times, six times**
64 przez ostatn[ie (0.5) półtora roku
**over the la[st (0.5) six months**

Although T's question is grammatically clearly addressed to W (line 54), H preempts W from answering by repeating and reconfirming his earlier “defense” of his wife. He downplays the number of critical events (“*only few times*”) and emphasizes that his wife was never “*hitting the children with a belt on a daily basis.”* The wife responds to this remark with a laugh particle (line 62), which she produces while being close to crying. Finally (lines 63–64), H gives an estimate of the frequency of the complained-about behaviors. Through his turn (from line 57), he has created a context where “*five times, six times*” is offered as a low, not high, number.

To sum up, in Extract 1, after W has cautiously complained about the problematic division of parental roles in the family, H started to describe his wife's rejective behavior toward their children. Although the content of his critique is grave (“hitting the child”), he conveyed his complaint in a considerate way by hesitating, using mitigated descriptions and oblique references, and downplaying the amount and severity of his wife's problematic actions. He avoided direct depictions of his wife's wrongdoing and focused instead on his own painful reactions and the children's perceptions. For all his complaints about his wife's behavior, H showed affiliation to her. During the complaint, there were moments when the complainer (H) and the complaint target (W) in different ways displayed togetherness and presented themselves as a team or one social unit.

### 4.2 Couple 2: being lonely while being together

*The next couple, in which the wife complains about her husband's unacceptable behavior, is quite like Couple 1 insofar as the activity of complaining involves balancing between displays of discontent of the spouse's wrongdoing on the one hand and mitigative solidarity, maintaining elements, on the other. The balance is tilted somewhat more toward mitigation and solidarity in Extract 1, as compared to Extract 2, but in each case, discontent as well as solidarity are present.. The transcript is taken from that part of the consultation in which T explores the couple's problem. H started his actual talk about problems by describing his wife's complaints about his reluctance to talk to her as well as about so-called “silent days” (a routine of spouses not to talk to each other for many days). Just before the segment presented below, T suggested that the time has come for the W to describe the problematic marital issues. Thereafter, T asked W directly:*

**Extract 2a**

01 T:Pani jak widzi problem °wasz°
**how do you(f) see °your(pl)° problem**
02 W:yyyy no ja:: yyy w naszym małzeństwie: yyyy uhmm
**well I:: uhm in our marriage: uhmm**

After some hesitations (line 2) and a pause of 2 s, W formulates in a decisive and undoubtful way the problem in her marriage:

**Extract 2b**

04 problem jest je↓den y taki ze: ja po prostu
**there's one problem that: I simply**
05 cały czas jestem samo↑tna (.) we dwo↓je
**I'm lone↑ly all the time (.) while being toge**↓**ther**

With her formulation “*in our marriage (...) there is one problem*,” W marks the centrality and omnipresence of the problem and signals that something essential is about to come. When she continues and formulates the marital problem from her perspective, she uses declarative statement by saying: “*I'm lonely all the time while being together*” (line 05). In that statement, her husband is mentioned only indirectly as somebody who is part of the togetherness, in which the wife feels lonely.

The completeness of this formulation is not only marked by a falling intonation and by T's ensuing confirmation token but also by W's subsequent gazing at her husband. Gazing at her husband shows that not only T is the recipient of W's problem formulation but that her turn is also addressed to her husband who is sitting next to her. By turning her gaze to her husband, she transforms her utterance from a simple propositional statement into a relational message. W formulates the core problem of her marriage. The following elaboration of her complaint (lines 08–19) then delivers the justification of her strong and decisive formulation of the central problem in the marriage and of her suffering.

**Extract 2c**
08 W:.hh maz yyy .hh na poczatku naszego małzeństwa
**.hh husband uhm .hh at the beginning of our marriage**
09 w ogóle ze mna nie rozmawi↑ał na takie tematy
**didn't ↑talk to me about certain topics at all**
10 ponizej yyy po- powyzej pewnego pułapu (.)
**below uhm ab- above certain level (.)** *((W moves her hand horizontally))*
11 pewnego pułapu powiedzmy informacji wymiana informacji=
**some level let's say of information exchange of information=**
12 T:=taki[ch biezacy]ch
**=the [everyday topics]**
13 W:[na tym pie-] bieza↓cych(.)
**[on this pie-] every↓*day* (.)**
14 natomiast jezeli byłby jakieś spiecia jakie yy ze
**on the other hand if there were some tensions such erhm that**
15 jezeli były jakieś problemy i dochodziło do do yy to
**if there were some problems and it came to to erhm then** *((W gestures))*
16 po prostu: po jakimś czasie dochodziło do pewnego::
**simply: after some time it came to some::**
17 momentu ze:
**moment that:**
18 (1.5) ((W imitates explosion with a hand gesture))
19 był wy↓buch
**it exp↓loded**

W starts to further elaborate on her husband's problematic behavior (lines 08–13) by describing the history of their marriage. In her elaboration, she uses certain descriptive devices (line 09) such as negative observation (Schegloff, 1988) “*he didn't talk to me about certain topics*” and the extreme case formulation “*at all*” (Pomerantz, 1986). Her account comes to a description of gradual consequences which her husband's behavior engenders: An accumulation of tensions (line 14–15) that leads to a final explosion (line 19). The emphasized expression “*it exploded*” conveys, together with its gestural illustration, the intensity of problems in the marriage. Despite the dramatic depiction of the central marital problem, it is surprising that it is delivered in an agentless manner (Here, an interesting parallel to Extract 1 can be observed, as there—see Extract 1a, lines 9–11—the husband spoke about “*strictness*” and “*rejection*” without explicitly attributing them to the wife.). With her expression “*it exploded*,” W depicts the event as a kind of chemical reaction in the marriage, thereby avoiding the identification of the person who exploded. The agentless account is picked up by the therapist, who asks W (line 20) who the agent of the explosion was—she or her husband? It seems that T does not accept W's cautious mode of agentless complaint but insists on a clearer picture of the event.

**Extract 2d**

20 T:eh kto w[ybuchał ] pa↑ni czy m↓az
**who was ex[ploding ] y↑ou or hus↓band**
21 W:[eksplozja]
**[explosion]**
22 (0.5)
22 W:eeeeee hhh no wygladało to tak ze: ze:
**eeeeee hhh well it looked that in the way tha:t tha:t**
23 ja chciałam zeby on ze mna rozmawi↓ał a on
**I wanted him to talk to me and he**
24 po prostu yyy (.) mm yyy a on po prostu ze mna
**simply erhm (.) mm erhm and he simply** *((H grunting))*
25 nie rozmawiał tylko zabierał kurtke i wycho↓dził
**wasn't talking to me he was just taking his coat and lea**↓**ving**
26 T: **mhm**

T's question shifting the format of talk from agentless in interpersonal does not immediately stop her agentless way of talking. In her account, W is setting up a contrast between her plausible and “normal” need of talking to her spouse and her husband's obvious strange response of leaving the house. Contrast structures are typically used in verbal interaction to depict someone's behavior as non-normal or at least inappropriate (Smith, 1978). In W's description, a contrast is constructed between her wish, i.e., her own inner world, and H's observable behavior, for which a concrete detail (“*taking his coat*”) is provided, which serves as “empirical” validation of her version.

**Extract 2e**

27 W:(.) i tak to wygladało (1.5) od momentu kiedy:
**(.) and it looked like that (1.5) since:**
28 weszliśmy do domowego kościoła <musimy> ze soba
**we joined the domestic church we < must>**
29 rozmawiać bo to jest nasze zobowiazanie
**talk to each other because this is our obligation**

After the therapist's minimal confirmation token (line 26), W indicates that the marital problem she was describing occurred at a certain period of their life, after which a turning point happened: “*it looked like that until*” (line 27). The event which marked the turning point was the couple's joining the domestic church, with which the obligation to communicate with each other came along. However, this obligation only partly solved the couple's main problem. In the continuation of her talk (lines 31–38), W comes up with a new complaint about her husband, who treated these couple talks as pure obligation (line 32), something that just needed to be done (line 38). Moreover, again, W uses the contrast format to mark the difference between her husband's attitude and her own experience: “*it helps me*,” “*I want that*” (line 38).

**Extract 2f**

31 W:.hh ale:: hhh yyyy małzonek podchodzi do tych
**.hh but hhh uhm my spouse's approach to these**
32 rozmów ta:k ze ze on to robi (.) bo musi (.)
**talks i:s that that he's doing them (.) because he must (.)**
33 to nie jest (.) bo (.) inna rzecz jest taka .hh
**it isn't (.) because (.) it's quite different from .hh**
34 ze ja rozmawiam bo faktycznie wiem
**that I talk because I actually know**
35 ze mi to po↑maga (.) wiem ze tego ch↑ce
**that it he↑lps me (.) I know that I wa↑nt that**
36 T:**mhm**
37 W: .h a inna rzecz jest jezeli ja to musze zrobić (.) yy
**.h and it's quite different from that I must do this (.) uhm**
38 załatwić to (.) i z gł↓owy (.) mam to za soba
**to have it done (.) and get it over with (.) to leave it behind me**
39 T:a skad pani wie ze maz to robi bo mu↑si (.)
40 **and how do you(f) know that your husband does it because he must (.)**
mówi pa↑ni czy pani to czu↑[je]
**does he tell y↑ou(f) or do you(f) f↑eel it**
41 W:[ta]k powie↓dział
**[he sa↓id so]**
42 T: **aha**
43 W:tak powiedział ze ze po prostu za kazdym razem
**he said so that that just every time**
44 kiedy on yy s siada do tej do do tego stołu
**when he uh s sits to this to to this table**
45 do tej świecy to (.) no to to jest dla niego
**to this candle that (.) well this this is for him**
46 yy nie do przeskoczenia to jest dla niego
**uh impossible to bear for him**
47 takie trudne
**so difficult**

Answering T's question, W quotes her husband, saying how difficult it is for him to talk (lines 43–47). In her answer, the character of W's account changes, becomes less critical, and shifts to an understanding of the reasons for her husband's behavior that was, a few turns earlier, the object of her complaint.

**Extract 2g**

48 T:a dlaczego maz musi to °robić°
**and why must the husband °do° that**
49 W: (0.8)
50 W:£hhhh ha ha (0.3) .hhh @dla mnie@ he he [he he ha ha ha£ £
**hhhh ha ha (0.3) .hhh @for me@ he he [he he ha ha ha**£
51 T:[dla pani [for you miss

T half-jokingly challenges W's moral perspective by inquiring about the reason for H's obligation to talk to her (line 48). In her response, W starts to laugh and answers “for me” (line 50), possibly realizing the paradoxical nature of her complaint. H joins his wife's laughter, which shows his emotional affiliation with her at this moment.

In summing up Extract 2, upon T's invitation to describe the couple's problems, W came up with a complaint about her husband's long-standing problematic behaviors. Her complaint initially included a description of her suffering; however, in the continuation of her account, she depicted in some detail her husband's unacceptable manners that have lasted through the time of the marriage. After the explicit description of her husband's wrongdoing, the W, in response to the therapist's question, showed understanding of the H's motivation. Thereby, W's complaint developed into a more considerate direction. Moreover, even though W's complaint sequence involves serious matters, it ended in a positive affective atmosphere with W's laughter and H's simultaneous smiling, which protects H's face.

### 4.3 Couple 3: my wife is afraid of a quarrel

*Whereas in Extracts 1 and 2, there are moments during which the couple displayed togetherness and performed as one social unit, such a sense of solidarity and cohesiveness is pretty much missing in the following case. There are signs of affiliation, though, but they are rare, they are only shown by the wife, they are unevenly distributed. The deep split between the spouses becomes visible, particularly when the husband is rounding up his critique of his wife by not just complaining about her behavior but in the form of characterological blaming* (Janoff-Bulman, 1979).

*In the following, two segments will be discussed, which include two complaints, one from W and a subsequent complaint from H. At the beginning of the session, the therapist tries to elicit from the resistant husband the reasons for attending therapy. Using the opportunity to respond, the wife begins to formulate a list of problems, albeit in a somewhat vague and inconclusive manner, such as “problems in communication,” “tension in the house,” and “anxiety about the wellbeing of their child”; she also reveals her own troubles such as “insomnia,” “stomach problems,” and “exhaustion.” Subsequently, the therapist asks W to clarify her description and asks, “Well at this point what is your guess? Is it-”*

**Extract 3a**

01 W:Domysł jest taki ze:
**My guess is tha:t**
02 na pewno bardzo sie róznimy z mezem (.) temperamentami=
**for sure we are really different (.) temperamentally=**
03 =ja jestem y- wrazliwa osoba raczej spokojna, powolna=
**=I am uhm a sensitive person rather calm, slow=**
04 =co tez denerwuje meza .h
**=what also irritates husband .h**
05 natomiast maz jest szybki j- no jest zaradny zyciowo
**whereas husband is fast i- well is resourceful in life**
06 wszystko faktycznie wszystko ogarnia robi zarabia .h
**everything actually everything gets done works earns .h**
07 tutaj nie ma w ogóle nic do zarzucenia (1.0)
**there's nothing wrong to be said (1.0)**
08 natomiast yy no jest je- jest bardzo yy .hh yy (3.5)
**on the other hand uhh well he i- is really uhh .hh uhh (3.5)**
09 £energiczny bym powiedziała£ yyy
**[£energetic I would say£ uhhh** ***[((smiling))***
10 i nie- nie zawsze: yy potrafi yy panować nad słowami
**and he can't always:s uhh control uhh his words**
11 i nad gestami .hh wiec yy
**and his gestures .hh so uhh**
12 T: co to znaczy? yy yy nie zawsze potrafi panować nad słowami
**what does that mean? uhh uhh he cannot always control his words**
13 i gestami?
**and gestures?**

In her response to T's question, W starts to describe what she sees as her husband's problematic behavior. She does not do this in a straightforward manner but approaches her problem formulation gradually and with various caution markers, such as hesitations, pauses, qualifications (“*I would say*”), and a lexical item (“*energetic*”) whose meaning is ambivalent: it can be a positive assessment of H, but can also be heard as a euphemistic expression for a disapproved habit, e.g., aggressiveness (lines 08–09). In the continuation of her description, she uses a negative observation (”*he can't control*“) to describe her husband's lack of self-control. She qualifies and mitigates her account with two additions: with her remark that her husband is “not always” in control, she implies that at times he indeed is in control of himself; and she limits his lack of self-control to words and gestures (lines 10–12). W's last formulation is immediately taken up by the therapist, who asks W to detail her condensed formulation.

Upon T's question, W starts to specify the description of her husband's behavior.

**Extract 3b**

14 W: to znaczy no:, bardzo czesto sie irytuje złości i yy yy
**it means we:ll, very often he gets irritated angry and uhh uhh**
15 mówi wtedy przykre rzeczy i takie atakujace raniace
**then he says unpleasant things and so attacking hurtful** ((licking her lips))

Again (line 14–15), W uses hedges (“*it means, well*”), qualifications (“*very often”*), hesitations (“*yy yy”*), the rhetorical figure litotes (“*unpleasant”*), and other politeness markers through which her critical characterizations of her husband' acting (“*angry*,” “*attacking hurtful*”) are mitigated. Despite her complaint, she acts considerately and seems to protect her husband by attenuating the severity of his behavior.

In the ensuing talk, T disregards W's account of her husband's behavior and draws W's attention instead to her perception of her husband's acting:

**Extract 3c**

16 T: i te (.) w tych (.) wtedy pani sie zapytuje czy .hh pani jest
**and these, in these, then you (f) wonder whether .hh you're**
17 nadwrazliwa czy to maz jest nadmiernie=
**oversensitive or is it husband that is overly=**
18 W:=tak=
**=yes=**
19 T:=agresywny, tak?=
**=aggressive, right?=**
20 W:=tak
**=yes**
21 T:Czyli nie ma pani jakby rozstrzygniecia
**That is you somehow don't have the conclusion**
22 W: To znaczy generalnie czuje yy .h stałe takie napiecie
**I mean in general I feel uh .h this constant tension**
23 i yy zastanawiam sie po prostu cały czas
**and uh I just wonder all the time**
24 >Zreszta, maz to moze potwierdzi< ze co chwile sie .h go pytam
**>Besides, husband can confirm < that all the time .h I ask him** < —((hand gesture toward the husband))–>
25 czy jesteś zły czy coś zrobiłam nie ta:k.
**are you mad have I done something wro:ng**.
26 ***((W's account of her feelings continues, describing that she feels husband's hostile attitude toward her, that she feels judged negatively and as if bothering him all the time)).***

In T's question, the source of the marital problems is located either in the wife (“*oversensitive”*) or in the husband (“*overly aggressive”*). W's confirmation is ambivalent, and she does not take sides and thus evades the answer; T explicitly formulates W's undecidedness in her response (line 21). As W continues her account (lines 22–26), she describes her own irritation and anxious thoughts (rather than her husband's behavior) and invokes her husband's view (line 24: “*husband can confirm”*). She also describes that she is torturing herself with the thought that she herself might be the source of the problem. In sum, while W's complaints about her husband are severe and definitely exceed a normal marital dispute, she protects him and shows consideration for him in several respects. She avoids hurtful expressions and uses euphemistic formulations in describing his behaviors, and she indicates self-doubts and addresses the possibility that she herself may be partly to blame for the couple's problem.

After W has completed her account, T turns to H and invites him to comment on his wife's statement (transcript not included). H starts his answer (Extract 4a, line 10) by declaring that he was prepared for this issue to come up and that he was expecting even prior to the session that this marital conflict would become a topic in therapy (“*when we come here there will be for sure will be about this conversation”*). Then, he moves on to the points which T has raised (lines 13–16):

**Extract 4a**

10 H:Znaczy generalnie właśnie myślałem
**I mean in general I was just thinking that**
11 jak >>przyjdziemy tutaj < < to bedzie
**when >>we come here < < there will be**
12 na pewno bedzie o tym yyyhmmm rozmowa .hh
**for sure will be about this uuuhmmm conversation .hh**
13 y: i tak sam sam myślac o tym no doszedłem do wniosku
**u: and by thinking by myself I came to the conclusion**
14 ze po prostu moja zona sie boi kłótni= .hh
**that simply my wife is afraid of a quarrel= .hh**
15 T: **=aha=**
16 H:=nie lubi: wyrzucać wszystk- z siebie .hh e- emocji
**=doesn't like throwing everyth- out .hh e- emotions**
17 T: **=aha=**

H describes his wife as someone who is unable or unwilling to face conflict situations (line 14); additionally, he characterizes her as not an open person who prohibits herself from expressing emotions. He portrays himself in contrast to his wife as an extrovert person who does not restrain his feelings and who acts in an expressive and vivid manner (line 18–19):

**Extract 4b**

18 H: y:: nie wiem no podnosze głos zaczynam gestykulować,
**u:: I don't know I raise my voice I start to gesticulate**
19 czy nie wiem .hh po prostu: widać ze ze jakieś takie
**or I don't know .hh simply: it's visible that that some**
20 skrajne emocje mna: targaja .hh
**extreme emotions are tormenting me .hh**
21 to (.) natychmiast jest e:: jakaś taka o: taki odzew
**then (.) immediately there is e:: some some o: such a response**
22 ze (.) czemu jezdzisz po mnie?
**that (.) why are you bum-rapping me?**
23 To [jes]t takie w cudzysłowiu tak?
**This is like in inverted commas ok?**

H claims that his behavior can evidently be seen and understood (“*it's visible”*) as the outward manifestation of an inner “*torment*,” but that his wife is not interested in this background and that she has no understanding of the reason for his agitated way of acting. On the contrary, he complains with the evidential source of a semi-quote (lines 22–23) that she accuses him of mistreating her, using the “why did you”-question format as a typical device for the construction of a reproach (Günther, 1996).

**Extract 4c**

25 H:czy (.) no ale to (.) >>to jest to jest < < główny problem .hh
**What (.) well but it (.) >>this is this is < < the main problem .hh**
26 ze ze ja nie dam rady po prostu
**that that I won't be able to just**
27 ro- rozmodlić sie w tym momencie i sie zamknać .hh
**start praying in this moment and to shut up .hh**
28 nie nie (0.5) nie uzewnetrzniajac prawda? .hh
**with no no (0.5) no externalization right? .hh**
29 nic właściwie bo to chyba o to chodziło by
**anything actually because this I guess that's what it is about**

Although H concedes that his behavior is a possible cause of the marital problems, he does not present himself as the one who is responsible. Instead, he rejects the expectations—implicitly attributed to his wife—to stay calm and to control his demeanor, which he describes ironic-sarcastically through an unrealistic exaggeration (“*start praying”*) and a vulgar formulation of obeying the order to stay silent (“*shut up”*). At that point, at which H is still talking to T, W is taking the turn and addresses her husband directly:

**Extract 4d**

30 W:nie nie to m:: bardziej mi chodzi nie wiem
**no no I mean more I don't know**
31 ze mógłbyś w- właśnie w jakiś taki zwiezły
**that you would actually in some concise**
32 trafny sposób y: nieraniacy y: formuł [ować co masz do mnie
**accurate way u: not hurting u: formulate [what you have against me**
33 H:[Iść pobiegać na przykład
**[go jogging for example**
34 no ale y: jestem za leniwy albo nie wiem
**but u: I am too lazy or I don't know**
35 nie mam siły albo nie mam czasu
**I don't have energy or don't have time**
36 **(4.0)**

W strongly disagrees with her husband, requesting that he formulate his critique more precisely and in a decent way (line 32: “*not hurting”)*. However, H continues his ironic-sarcastic line (“*go jogging”*) and mockingly gives the blame to himself (lines 34–35).

In sum, in Extracts 3 and 4, two interlinked complaints can be observed. W first complained about her husband's irritable and aggressive behaviors, where after H complained about his wife's inability or unwillingness to understand and tolerate his emotions. The spouses' complaints were performed quite differently. W described her husband's behavior in a resolute yet considerate way, expressed doubts about her perception, and was even open to self-blaming. She displayed affiliation with her husband and treated the couple as one social unit whose malfunctioning can be repaired. In contrast, H took a thorough confrontative stance toward his wife. He took his wife's suggestion to interact in a more friendly manner as a restriction of his freedom of expression. He furthermore accused his wife of being unable—or unwilling—to see his inner ordeal. In his view, the couple's marital problems were first and foremost his wife's problems. An additional outstanding difference between the spouses' complaining practices is that W was complaining about her husband's behavior, whereas H's complaints were directed at his wife's personality and character.

### 4.4 Couple 4: “you are lying”

*Whereas Extracts 3 and 4 were characterized by H's unidirectional hostility toward his wife, in the following case, both spouses directly and aggressively express their accusations. The intensity of their mutual hostility becomes manifest in the fact that they stop to talk to the therapist and turn directly at each other with repetitive blaming. Mutual blame and denial seem to emerge with remarkable rapidity on the part of both partners. An illustrative example of this process is given below. Just before the following segment, H presented himself as involved in family matters*.

**Extract 5a**

01 W: teraz to kłamiesz Romek nie chciał iść na: urodziny erm
**now you're lying Romek did not want to go on birthday party erm**
< -*making an eye contact with the therapist and redirects her gaze to the husband shortly after*
02 nie chciał iść na komunie swojego chrześniaka .h
**did not want to go to his godson's communion .h**
03 bo powiedział ze tam nie jest potrzebny dopiero go prosiłam.
**because he said he wasn't needed there only then I asked him**.
04 (0.5)
05 H:To juz wymyślasz ter[az.
**Now you're making thi[ngs up**.
06 W:[Nie Romek. [Tak było.
**[I'm not Romek. [That's how it was**.
07 H:[To juz jest kłamstwo.
**[This a lie**.
08 W: Tak było Romek.
**That's how it was Romek**.
09 H:To juz jest kłamstwo.
**This is a lie**.
10 W: W pierwszy dzień świat tez ze mna nie poszedłeś
**First day of holiday you also didn't go with me**

W, strongly disagreeing with her husband's statement, directly turns to him and says, “*now you are lying*.” After that, she starts describing his behavior in the third person (lines 01–03). The addressee of this part of the utterance is T, and this re-direction is emphasized by W, who performs a brief eye contact with him, and shortly after doing so, she redirects eye contact to her husband while maintaining the third-person description in her utterance. It thus becomes clear why the next turn of speech is taken by the husband (line 05) and not by T. At this point in the conversation, a series of overlapping turns begins during which the spouses take extremely opposing positions; their exchange is an extreme example of antagonistic stance (Dersley and Wootton, 2000). The spouses use repetitions: “*that's how it was*” and “*this is a lie*” with increasing vigorousness to make their opinions clearer and stronger. These mutual accusations are made in the lexical form of unambiguous indicative sentences (lines 06–09), after which W, without direct interference from her husband, continues with another argument for her husband's lack of involvement in family matters (line 10). This statement is again countered by H some turns later (transcript not included).

An exchange like that is continued until interrupted by the therapist with a question “*what are you doing right now? Are you trying to come to an agreement?*” (transcript not included). One might think that taking the conversation to a meta-level (to start communicating about the communication) might stop the mutual blaming, but the conversation takes a different turn. Therapists' question invokes an exchange that can be seen as producing arguments on the meta-level of communication as presented below.

**Extract 5b**

32 H: ni[e:: (.) wygrać (.) to kto ma wiec- wieksze atuty::
**no[:: (.) to win (.) it who who has stro- stronger assets::**
33 T:[czy:::: czy właśnie-
**[or:::: or just-**
34 H:i::: kto lepiej dalej I kto [do tyłu
**and::: who better further and who [backwards**
35 W:[nie:: kto jest biedniejszy:
**[no*::* who is more poor:**
36 H:i kto do tyłu [sie
**and who backwards [**
37 W:[nie nie kto jest bardziej poszkodowany:
**[no no who is more of a victi:m**
38 kto jest po prostu::: [bie:::dny::::::
**who is just::: [unfo*:::*rtunate*:::*:::**
39 H:[kto siegnie po mocniejsze argumenty
**[who will reach for stronger arguments**
40 do tyłu:: [w prze-
**backwards:: [into the pa-**
41 W:[nie:: [kto jest po prostu biedny i [posz-
**[no:: [who is just poor and [harm-**
42 H:[w przeszłość [w przeszłość
**[into the past [into the past**

In this part of the conversation, the spouses argue about what is the purpose of the conversation they are having. They both acknowledge that their conversation is a kind of performance in front of the therapist, during which they each seek to show a different aspect of how they and their relationship function. W accuses her husband of seeking to present himself as the unfortunate and more of a victim (lines 35, 37, and 41), while H points out that his wife bases her arguments on events from the distant past of their relationship (lines 32, 34, 36, 39–40, 42). T's question, which in principle was supposed to interrupt the sequence of mutual accusations, stops the spouses and makes them reflect on what is currently happening during the therapy session, actually became a trigger for another exchange of accusations, which is eventually crowned with a long statement of H (starting in line 42). This lengthy statement (stretching all the way to the line 68; only fragments are included below) contains many accusatory elements.

**Extract 5c**

43 H:bo:: y::::: tutaj e::: ja na przy- pod tym katem
**because:: u::::: here er:::m I'm near- from this perspective**
44 jesteśmy TOTALNIE inni: ja mam (.) ((click)) (1.0)
**we're TOTALLY different: I have (.) ((*click*)) (1.0)**
45 pamieć ta:↑ka:: (1.0) m ze::: >złe: rze↑czy::::< (.)
**su:↑ch:: memory (1.0) m that::: >bad: thi**↑**ngs:::: < (.)**
46 jakoś tak nie wiem tak mnie jakoś natura stworzy↑ła
**kind of I don't know that's how nature created me somehow**
47 ze złe rzeczy (.) mam wypierane.
**that bad things (.) are repressed from me**.
48 (1.5)
49 ja naprawde złych rzeczy nie pamie↓tam. (0.5) (click)
**I really don't remember bad things. (0.5) ((*click*))**
50 bo bym dawno zwariował °jakbym to miał pamietać°
**because I would go crazy a long time ago °** **if I had to remember them** **°**
51 a moja zona jest biedna pod tym katem i h
**and my wife is miserable at this respect and h**
52 ja jej współczuje strasznie z całego serca za ↑to::
**I feel very sorry for her with all my heart for ↑it::**
53 bo tak naprawde moja zona pamie↑ta TYLKO złe rzeczy
**because my wife actually remembers ONLY bad things**

H stresses with particular emphasis (making an eye contact with T, saying the word “*totally*” louder, using hands in an emphasizing manner) on the differences between the spouses in terms of “*remembering*” or “*not remembering*” the past situations (line 44). First, he presents himself as someone who does not pay attention to experienced past wrongdoings. He is doing that by giving an undisputable account of his inability to remember bad things: “*that's how nature created me somehow*” (line 46). Next, he produces a passive voice sentence as if he did not have any control over his mind (line 47). He also reaches for extreme formulations stating that he himself “*would go crazy a long time ago*” if he was doing the same thing as his wife (line 50). Then, he continues with the presentation of his wife's qualities (line 51), portraying her as miserable or suffering and expressing his sympathy toward her (line 52). Non-verbal activity of the wife (pressing her lips, looking away, and covering her face with her palm) suggests that she does not acknowledge this as a sign of affiliation or support. Lines 51 and 52 could be read as an attempt at fake affiliation made with irony. After that, H uses another extreme case formulation (“*my wife actually remembers only bad things*”), which again serves the purpose of contrasting their ways of “remembering things” and legitimizing the complaint.

In sum, Extract 5 is the most inconsiderate case of unmitigated marital hostility and antagonistic mutual complaining. The spouses do not talk about themselves; instead, they focus on the other's wrongdoings. Moreover, since they do not back down but insist on their positions and versions of past events, their conversation shows that their marital communication is deadlocked. The object of their complaints is not just a single act or event but comprises the entire person of the other and becomes, thus, a characterological blaming (“*we are totally different*”). Moreover, the aggressive complainers, when defending themselves, adopt a meta-perspective and resort to irony and sarcasm, thus demeaning the complaint's target.

## 5 Conclusion: the spectrum of complaints

In our exploration of complaint sequences in couple therapy first consultations, we observed a great variation of different ways and modes of complaining. However, this variety of complaint practices that occurred across couples and spouses is by no means chaotic and fortuitous. Complaints can be arranged along various components, but given the triadic constellation of our study object, the most pertinent dimension for the ordering of complaints is the level of consideration the couples showed when talking about the marital conflicts and the problematic behaviors of their respective spouses.

Based on a set of modes and policies, the various complaining practices can be arranged on a spectrum at one end, which is what we will call “considerate complaining,” and at the other end, there is offensive or “inconsiderate complaining.” The couple that was shown in Extract 1 was characterized by the most cautious way of complaining, whereas the complaining practices of the couple in Extract 5 were the most offensive and unmitigated. Extract 2 was close to Extract 1, yet not as cautious as it, whereas Extracts 3 and 4, which were characterized by an asymmetry of hostility between the spouses, leaned toward Extract 5. Taken together, different complaint sequences can be arranged as a spectrum of complaints.

Based on our empirical analysis, three components can be distinguished by which complaints can be constructed as more or less considerate resp. inconsiderate: object, mode, and (dis-)affiliation.

How a complaint makes reference to its object can vary significantly: when the complaint is made considerately, its object—the alleged infringement—is usually left implicit and only referred to with paraphrases, allusions, or euphemisms (“*energetic*”); in contrast, the object is identified and named explicitly when the complainer does not show consideration for the complaint target (“*you are lying*”). Furthermore, a considerate complaint is usually limited to the specific conduct of the complaint target, while an inconsiderate complaint focuses on the target's entire person and character. Moreover, cautious complaining is very often done with a focus on the suffering of the complainer's self, whereas reckless complainers mostly focus on the complaint target and his/her wrongdoing.

Mode refers to the specific ways and forms in which a complaint is communicated. In general, it can be observed that complainers who act regardful are solution-oriented and keep the integrity of the couple in mind, in contrast to ruthless complainers who are blame-oriented throughout their actions and care less about safeguarding the couple. It can further be observed that in the offensive mode, complainers often switch modality and turn to irony or sarcasm, whereas considerate complainers do not change modality and stay in the serious mode of matter-of-fact talking. Moreover, considerate complaints about the spouse are usually addressed to the therapist, whereas offensive complainers tend to turn directly to the target of their complaints (Here, the complaint may take on the character of an accusatory attack).

The location of a complaint at the complaint spectrum is furthermore determined by the complainer's affiliation or disaffiliation with the spouse as the complaint target. When a complainer acts considerately, she/he acknowledges the spouse's vulnerability and exercises caution to protect the other's self-image. Considerate complaining also implies that the complainer displays a continuing commitment to the couple as a social unit and shows an interest in finding common ground. In contrast, we call inconsiderate complaining when the complainer is dismissive about the vulnerability and self-image of the other and obviously does not care much about the marital relationship.

The following chart (see Table 1) may give a synopsis of the multi-dimensional dichotomy of activity patterns between which the spectrum of complaints stretches.

**Table 1:** The spectrum of complaints.

**Considerate complaining**	**Inconsiderate complaining**
**Object**	
Complaint object is expressed implicitly	Complaint object is expressed explicitly
Complaint object involves specific behaviors of the spouse	Complaint object involves the character of the spouse
Complainer is focusing on the suffering of his/her self	Complainer is focusing on the wrongdoing of the other
**Mode (Modality)**	
Complainer is solution-oriented	Complainer is blame-oriented
Complainer stays in the modality of serious talk	Complainer unilaterally switches to other modalities such as irony or sarcasm
Complaint is delivered in talk to the therapist as recipient	Complaint is delivered directly to the target, blending into blaming
**(Dis-)Affiliation**	
Complainer acknowledges the other's vulnerability and exercises caution to protect the self-image of the other	Complainer is dismissive about the vulnerability and self-image of the other
Complainer displays commitment to the relation and treats the couple as a social unit	Complainer is uncaring and does not show an interest in the relation
Complainer endorses the perspective of the other.	Complainer is dismissing or devaluing perspective of the other.

Features at the respective endpoints of the spectrum are logically related to each other, and, in fact, they often co-occur, thus forming a kind of considerate or inconsiderate “complaining pattern.” However, this need not always be the case. The components are not invariably tied to each other and can occur in various combinations. For example, it can be observed that in the delivery of a complaint the considerate and offensive mode may alternate, such that a blaming is followed by an understanding or an accusation is mellowed by a subsequent account.

## 6 Discussion

### 6.1 Conceptual implications

It is a key contribution of our study that we have ordered practices of complaining along a spectrum according to their degree of considerateness. Earlier, CA research on complaining has primarily focused on distinct practices that constitute utterances as complaints (such as extreme case formulations, negative observations, or litotes formulations). The spectrum of complaints we have shown in this article complements the results of earlier research with a more holistic view of complaining in one setting. It is, however, important to bear in mind that our findings come from a triadic framework, and they may not apply to other kind of settings. However, based on our observations, the question arises whether other social activities are gradable.. So far, research in conversation analysis has not dealt with this question. Several studies have introduced contrastive conceptual schemes for the description of specific interactional phenomena, e.g., the opposite mode of embedded or exposed correction (Jefferson, 1987) or the distinction between offering and requesting assistance (Kendrick and Drew, 2016). We think that the concept of a “spectrum,” which we introduced, would allow for a more nuanced view of various interactional phenomena and would provide a more realistic picture of the social world.

A further implication of our study pertains to the concept of “face.” Complaining about a co-present spouse is what Goffman (1955) called a face-threatening act. It is evident that by bringing up complainable matters in the spouse's behaviors or character, the complainer invokes a threat to the spouse's face. Yet, the complainer's own face is also at stake. Complaining about co-present others is generally considered as something to be avoided, and potentially as an indication of a problem in the complainer's own character. The specific setting of couple therapy begs the question of how the practices of “face work” which a couple has developed and practiced over time in the intimacy of their togetherness, are reproduced or altered in the presence of a third observer.

Goffman discusses how potential or actual face threats are mitigated in interaction. Face-threatening topics or actions can be avoided, or if they occur, they can be made ambiguous, blended with displays of respect, or in other ways smoothened (Goffman, 1955, p. 217–219; Brown and Levinson, 1987). Such smoothening is typical in considerate complaining, whereas inconsiderate complaining comes close to what Goffman called aggressive use of face-work. In couple therapy, each spouse needs to decide whether to respect the mutuality of the participants' concern for each other's face (which is typical for most ordinary interaction), or whether to score points to one's own face at the expense of the face of the spouse. How the spouses behave in this situation probably depends in no small part on how considerate or inconsiderate they perceive each other's actions to be. “Face Work” in couple therapy is thus a constellation of double contingency that needs to be taken into account.

### 6.2 Clinical implications

Exploring our database of nine initial consultations of couple therapy, we observed that, additionally to the differences between specific complaining modes and practices, there are also differences between individuals and couples. Some individuals tend to complain in certain ways and others in other ways and so do couples: some are prone to considerate complaining and others consistently choose to complain in an offensive way. Over the years of marital life and over a shared history of controversies and quarrels, couples obviously cultivate a certain routine or habit of complaining which they quite consistently practice and which they cannot easily abandon in the psychotherapeutic setting.

One possibility is that a couple's habit of complaining may be rooted in the personalities of the spouses. This assumption is supported by the results of the Shedler–Westen assessment procedure (Shedler and Westen, 2007) to which the study participants were subjected. In our small database, the offensive ways of complaining were associated with personality pathology—either narcissistic or borderline personality disorder—while the considerate type of complaining was associated with the absence of such a pathology. In the data presented above, the couples in Extracts 1 and 2 were diagnosed as having no personality pathology, whereas the two other couples had such pathology: in Extracts 3 and 4, the husband was assessed as having marked narcissistic personality traits, and in Extract 5, both spouses scored high in regard to disordered personality traits—narcissistic (husband) and borderline (wife). We should emphasize, however, that our limited data does not give evidence for a one-to-one relationship between personality and ways of complaining. As clinicians, we would rather expect that lack of personality pathology might be associated with flexibility on the part of the complainer in moving between different degrees of considerateness, while personality pathology might be associated with a more rigid way of complaining.

The idea of reflecting on how personality pathology can relate to conflict or discord in marital couples is not new and there is a body of research on this matter (e.g., Chen et al., 2004; South et al., 2008; Bouchard et al., 2009; de Montigny-Malenfant et al., 2013). In our study, we look from a different angle by emphasizing those aspects of patients' personality functioning that seem crucial from the perspective of the conversational practice of blaming and complaining. Our research shows that such aspects as the ability to mentalize, the ability to regulate emotions, make adequate attributions, and perceive causality, as well as the management of the threat to self and the need to defend oneself manifest themselves in complaining practices.

Mentalizing refers to the ability to understand beliefs, feelings, and motivations of the other and is postulated to be compromised in people with PD (Fonagy and Luyten, 2009). In couples therapy, it can express itself in the ability to take the spouse's perspective, concede his/her point, and manage the conflict accordingly. This ability was much more evident in instances of considerate complaining in our data.

The ability to mentalize is closely related to the ability to adaptively regulate emotional states (Schwarzer et al., 2021), which is another construct with clinical relevance and pivotal role in PDs. People who are emotionally dysregulated have difficulties in modulating, assessing, and expressing emotional responses in terms of their intensity, their maintenance, and their ending (Gross, 2014). There are several emotion regulation strategies that can be utilized by people experiencing intense emotions that vary in their level of adaptivity. In our database, it is observable that considerate complaints were accompanied by an effective kind of regulating emotions of the listening spouses.

The way couples were attributing the blame and how they related to the causality of problems at hand was also an important feature of the spouses' personality functioning. In the case of considerate complaining, complainers painted a much more complex map of the causes and circumstances behind someone's behavior and often acknowledged their role in co-creating the difficulty in question. In the case of inconsiderate complaining, it could be seen that the complainer often attributed the source of the problems to the “outside” and “blamed the other.” It is assumed that people who have difficulties with emotion regulation can experience distortions in the perception of the social context in which emotion is experienced, thus increasing the likelihood of using the defense mechanism of projection (Kaufmann et al., 2022) and inadequate assessment of reality.

All of the above is relevant to the functioning of the personality and its level of dysfunction and can be linked to the way of complaining observed during therapy session (see Table 2).

**Table 2:** The spectrum of complaints in relation to personality functioning.

**Considerate complaining**	**Inconsiderate complaining**
**Personality functioning**	
High level of mentalizing self and other	Poor mentalization, inability to keep others' perspective in mind
Effective strategies of emotion regulation	Complaint is accompanied by emotion dysregulation or ineffective means to regulate

### 6.3 Practical implications

In couple therapy first consultations, the clinician collects information that will help him/her to understand the couple's functioning and problems. It is obvious that the content of the spouses' talk—what they tell about their everyday life, difficulties, disappointments, and quarrels—is an important source of knowledge that facilitates the clinician's understanding. This information is delivered, to a large extent, in complaints. Yet, the fact that couples and individual spouses complain in such different ways suggests that the spouses' practices of complaining are an additional important source of information for the clinician. Considerate ways of complaining might suggest that there exists a firm ground on which processes of positive change can be built. In contrast, an inconsiderate way of complaining might suggest that the couple's problems are deeply rooted and that much work needs to be done to solve them. In sum, the way of complaining might give the clinician as much, if not more, information about the couple and their problems, than does the actual content of their complaints.

In this study, our analysis was primarily concerned with the composition and delivery of complaints in couple therapy sessions. Further studies are needed that focus on the other spouse's reception and response to a complaint and pay specific attention to the therapist's ways of dealing with a complaint.

## Data availability statement

The datasets presented in this article are not readily available because the dataset involves video recordings of couple therapy sessions. Such clinical materials cannot be shared with third parties. On request, we can provide anonymized transcripts of the sessions. Requests to access the datasets should be directed to bernadetta.janusz@uj.edu.pl.

## Ethics statement

The studies involving humans were approved by the BioEthical Committee, Jagiellonian University Medical College. The studies were conducted in accordance with the local legislation and institutional requirements. The participants provided their written informed consent to participate in this study. Written informed consent was obtained from the individual(s) for the publication of any potentially identifiable images or data included in this article.

## Author contributions

BT: data collection, data analysis, and writing the article. BJ: designing the study, data collection, data analysis, and writing the article. JB: data analysis, and writing the article. AP: designing the study, data analysis, supervision of data analysis, and writing the article. All authors contributed to the article and approved the submitted version.
